# Enhanced X-ray free-electron laser performance with optical klystron and helical undulators

**DOI:** 10.1107/S1600577524003254

**Published:** 2024-06-11

**Authors:** Christoph Kittel, Marco Calvi, Sven Reiche, Nicholas Sammut, Guanglei Wang, Eduard Prat

**Affiliations:** a Paul Scherrer Institut Forschungsstrasse 111 5232Villigen PSI Switzerland; b University of Malta MsidaMSD2080 Malta; RIKEN SPring-8 Center, Japan

**Keywords:** free-electron lasers, synchrotron radiation, undulators, polarization of light, X-ray lasers

## Abstract

This article presents a demonstration of the improved performance of an X-ray free-electron laser (FEL) using the optical klystron mechanism and helical undulator configuration, in comparison with the common planar undulator configuration without optical klystron. The demonstration was carried out at Athos, the soft X-ray beamline of SwissFEL. Athos has variable-polarization undulators, and small magnetic chicanes placed between every two undulators to fully exploit the optical klystron. It was found that, for wavelengths of 1.24 nm and 3.10 nm, the required length to achieve FEL saturation is reduced by about 35% when using both the optical klystron and helical undulators, with each effect accounting for about half of the improvement. Moreover, it is shown that a helical undulator configuration provides a 20% to 50% higher pulse energy than planar undulators. This work represents an important step towards more compact and high-power FELs, rendering this key technology more efficient, affordable and accessible to the scientific community.

## Abstract 2

This paper presents a demonstration of the improved performance of an X-ray free-electron laser using the optical klystron mechanism and helical undulator configuration, in comparison with the common planar undulator configuration without optical klystron. The measured improvements are compared with simulations and show a good agreement and a significant reduction in saturation length and an increase in saturation power.

## 1. Introduction

X-ray free-electron lasers (FELs) are state-of-the-art instruments for important scientific discoveries across many fields (McNeil & Thompson, 2010 ▸; Pellegrini *et al.*, 2016 ▸; Bostedt *et al.*, 2016 ▸). However, owing to the large footprint and high costs of such facilities, few exist so far, which limits their overall scientific impact. Thus, incentives are strong to develop and employ technologies which help reduce the overall footprint and attached costs of X-ray FEL facilities, so that we may enhance their potential scientific impact on our society. There is a widespread international scientific effort to construct more compact and cost-effective X-ray facilities. Examples of such initiatives are the CompactLight (D’Auria *et al.*, 2019 ▸), the ultra-compact X-ray FEL (Rosenzweig *et al.*, 2020 ▸) and the EuPRAXIA (Assmann *et al.*, 2020 ▸) projects. We also acknowledge the recent fundamental advances made on plasma-based FELs (Wang *et al.*, 2021 ▸; Pompili *et al.*, 2022 ▸; Galletti *et al.*, 2022 ▸; Labat *et al.*, 2023 ▸), relevant for projects such as EuPRAXIA.

The X-ray FEL radiation is produced by a high-brightness electron beam with an energy at the GeV level traveling through an undulator beamline. The FEL radiation wavelength can be expressed via the undulator resonance condition (Bonifacio *et al.*, 1984 ▸) 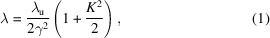
where λ_u_ is the undulator period length, γ the Lorentz factor of the electron beam and *K* the undulator field deflection parameter. The FEL radiation field grows exponentially until the process reaches saturation. The overall FEL performance is normally characterized by two quantities: the required length to reach saturation, also called the saturation length, and the FEL power at saturation.

In this article we show a reduction of the saturation length of an X-ray FEL by approximately 35%. Such a reduction of the undulator length is an important step for building more compact and affordable FEL facilities. For a given undulator length, a shorter saturation length allows reaching saturation at shorter radiation wavelengths and provides, for example, more space to increase the FEL pulse energy with undulator tapering (Kroll *et al.*, 1981 ▸) [*i.e.* changing the undulator field *K* to compensate the loss in electron energy along the undulator due to the FEL process so that the resonance condition remains fulfilled, see equation (1)]. The improvements are due to two effects. First, we employ a helical undulator configuration instead of the typical planar configuration, which corresponds to a stronger coupling between the electrons and photons during the FEL process. This, in addition to reducing the saturation length, helps to significantly increase the FEL saturation pulse energy. Potential disadvantages of helical undulators are that they are technically more complex than undulators with simple planar configuration and that in some cases planar polarization is essential for certain types of user experiments. Second, we use the optical klystron (OK) (Vinokurov & Skrinsky, 1977 ▸; Elleaume, 1983 ▸; Litvinenko, 1991 ▸), *i.e.* we speed up the FEL process using magnetic chicanes between the undulator modules to further reduce the saturation length.

A comparison of the FEL performance before saturation between helical and planar undulator configurations was made at FERMI in the extreme ultraviolet regime (Allaria *et al.*, 2012 ▸). It was shown for a radiation wavelength of 32.5 nm that the FEL gain length (the length required to increase the FEL power by a factor of *e* in the exponential regime) is improved from 2.5 m to 2 m when using helical undulators. The reduction of the FEL saturation length via the OK has been demonstrated separately at VUV and soft X-ray FELs (Penco *et al.*, 2015 ▸, 2017 ▸; Prat *et al.*, 2021 ▸). Prat *et al.* (2021 ▸) demonstrated that the OK reduces the saturation length between 15% and 30% for radiation wavelengths between 1 nm and 2 nm. Here we demonstrate, in both measurements and simulations, that the two enhancements can be effectively combined to reduce the saturation length by up to 35% for various radiation wavelengths in the X-ray regime. An initial empirical demonstration of both effects was presented in the Athos first lasing paper (Prat *et al.*, 2023 ▸).

**Figure 1:**
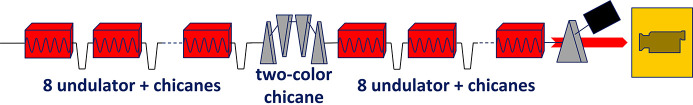
Schematic layout of the SwissFEL Athos beamline (not to scale), adapted from Fig. 1 in Prat *et al.* (2021 ▸).

The work was carried out at Athos (Abela *et al.*, 2019 ▸; Prat *et al.*, 2023 ▸), the soft X-ray beamline of SwissFEL (Prat *et al.*, 2020 ▸). It is the first FEL facility capable of providing fully variable undulator configurations, *e.g.* by offering the full *K* range for helical and planar polarizations thanks to the radial symmetry of the Apple-X module, as well as thoroughly exploiting the OK effect at the same time. A sketch of the Athos undulator is shown in Fig. 1 ▸. It consists of 16 Apple-X undulator modules (Calvi *et al.*, 2017 ▸; Schmidt & Calvi, 2018 ▸; Liang *et al.*, 2021 ▸), each 2 m in length with a period length of 38 mm, with the ability to provide variable undulator field and polarization. The deflection parameter *K* can be varied up to 3.7 in all polarizations. This, combined with the possibility to tune the electron beam energy between 2.9 GeV and 3.4 GeV, and assuming a minimum *K* of 1 for sufficient FEL coupling, corresponds to a radiation wavelength range between 0.65 nm and 5 nm (1.9 keV to 260 eV), see equation (1). The intra-undulator sections between the modules are 0.8 m long and house small magnetic chicanes. Besides acting as phase shifters, the chicanes can delay the electron beam by up to 8 fs (for an electron beam energy of 3 GeV), useful for implementing the OK, and/or add a horizontal offset of up to 300 µm (Prat *et al.*, 2016 ▸). In addition to the OK, the chicanes can be employed for other operational modes like high-brightness SASE (McNeil *et al.*, 2013 ▸) or to produce high-power and short FEL pulses (Prat *et al.*, 2015 ▸; Tanaka *et al.*, 2016 ▸; Wang *et al.*, 2024 ▸).

## 2. Experimental setup

We measured FEL gain curves for four different configurations: helical undulator configuration (positive circular polarization: C+) with and without OK, as well as planar undulator configuration (linear horizontal polarization: LH) with and without OK. In this way we can compare the impact of both OK and the undulator configuration. All configurations were measured under the same beam conditions for two different FEL wavelengths (photon energies): (1) 1.24 nm (1000 eV) and (2) 3.10 nm (400 eV). As detector for the pulse energy measurements the Athos photon-beam-intensity gas monitor was used, which is a device similar to the one installed in the Aramis beamline (Juranić *et al.*, 2018 ▸).

The measurements took place during one experimental shift under the same electron beam conditions (see Table 1 ▸). The electron beam energy was fixed at 3375 MeV, while the bunch charge was 200 pC. We adjusted the radiation wavelength solely by tuning the *K* parameter. With time-resolved measurements of the electron beam using an X-band RF transverse deflector we determined the bunch length to be around 25 µm full width at half-maximum, with an average peak current of around 2.4 kA and a slice energy spread of around 1.2 MeV at the bunch core. The normalized projected emittance at Athos is normally measured between 400 nm and 500 nm. From measurements at the hard X-ray beamline (Prat *et al.*, 2019 ▸) we expect a slice emittance between 250 nm and 300 nm at the bunch core in Athos. The optics were set to have an average β-function in the undulator of approximately 6 m.

The measurement series for each wavelength started with a set-up of the electron beam with the accelerator tuned for the maximum output in FEL pulse energy. This is mainly to ensure that the accelerator is operated with standard performance and to provide us with a good starting point for our measurements. For every wavelength we followed the same set-up and optimization procedure. The set-up with OK consists of the following steps for both polarizations: (1) We start without lasing conditions by randomly detuning the field of all Apple X undulator modules. Furthermore, we open the gaps of all chicanes in order not to apply any delays or offsets to the electron beam. (2) Starting with upstream modules, we tune their individual undulator fields to the desired wavelength, one module after the other, but only until we reach a stable gas-detector signal, usually around one microjoule to start above the noise floor. (3) We simultaneously scan the delays of the chicanes between the undulator modules currently in use and set the optimum value. (4) We add one undulator module immediately downstream, scan the delay of the additional chicane and set the optimum value. (5) We repeat step (4) until the optimal delay is found to be smaller than 0.1 fs. In this case we switch to search for phase matching conditions between the modules. (6) We add one downstream module and fine-tune the chicane between it and the previous module to reach their phase matching condition. (7) Repeat step (6) until FEL saturation is reached and the increase in FEL output from additional modules drops to below 10%.

For the case without OK (for both polarizations), the procedure is simplified. We start with steps (1) and (2) as described above, but with the difference that we then optimize individually the phase matching of the chicanes between the initially used modules [compare with step (3)]. We then continue with steps (6) and (7) as described for the OK case.

We started measurements with the helical undulator configuration at all radiation wavelengths. Upon switching to planar configuration we re-optimized the matching of the optics at the undulator entrance to adjust for the different focusing of the helical and planar undulator configurations.

## 3. Numerical calculations

We have simulated the measurements for all cases with the code *Genesis 1.3* (Reiche, 1999 ▸). As input parameters we used the measured or estimated electron beam parameters, and machine settings such as undulator *K* and chicane delays summarized in Table 1 ▸. We estimate the energy spread from the optimal delay of the first OK by utilizing the following expression (Ding *et al.*, 2006 ▸), 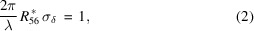
where λ is the FEL radiation wavelength, σ_δ_ is the uncorrelated energy spread and 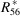 is the optimal longitudinal dispersion of the chicane, which is approximately twice the delay. The longitudinal dispersion is the variation of the longitudinal position of a particle as a function of its relative energy deviation with respect to the average energy of the beam. This equation shows that longer wavelengths require larger 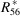, and thus longer delays. The energy spread values obtained with this equation fit reasonably well with the measurements performed with the X-band RF deflector. For the calculation we assume a flat current profile of the electron bunch of 2.4 kA, but with only 80% of the bunch length, and thus also charge, contributing to the FEL output. This restriction is made to match the global FEL output. It is justified because the current peaks at the edges of the charge distribution feature a higher emittance and lack overall quality for good lasing.

## 4. Results

**Figure 2:**
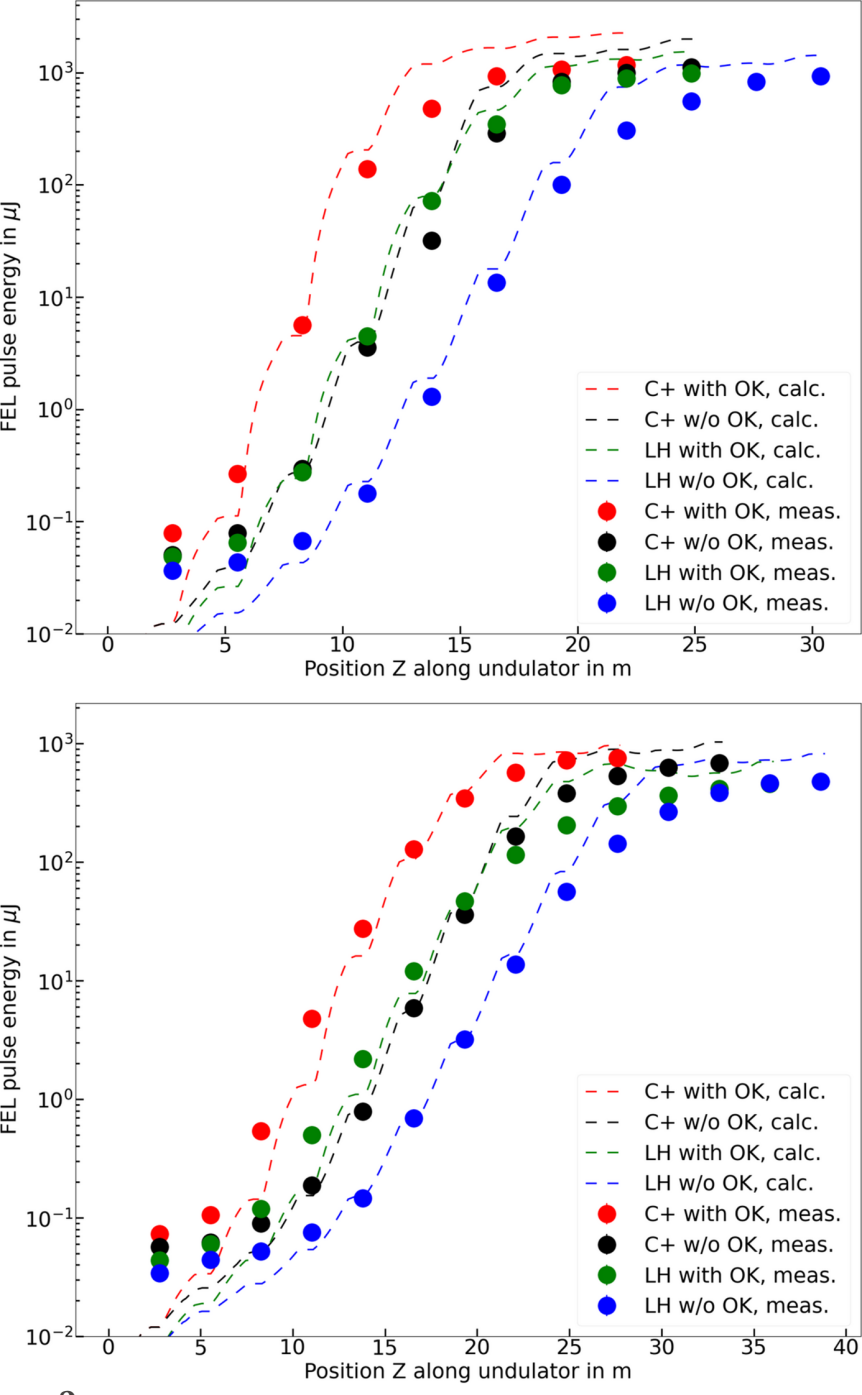
Measured (solids points) and simulated (dashed lines) FEL gain curves for all combinations of helical (C+)/planar (LH) polarization and with/without OK at two different radiation wavelengths: 3.10 nm (top) and 1.24 nm (bottom).

Fig. 2 ▸ shows the measured and simulated FEL gain curve results for the two radiation wavelengths and the four different configurations: planar (LH) with and without OK, helical (C+) with and without OK. The measurement data agree reasonably well with numerical calculations in the exponential regime, in which we are interested. We think that the discrepancies can mostly be accounted for by two effects. First, the simulations assume constant properties along the bunch, for instance the electron energy, peak current, emittance and the trajectory, while in reality these parameters may vary as a function of the longitudinal position of the electrons. In particular, the wakefields within the undulator can significantly change the electron energy along the bunch. Second, we have only limited knowledge of the electron beam distribution, and there may be calibration and alignment errors of various devices in the machine. We also acknowledge that measured values at around 0.1 µJ or below diverge from the simulation data due to the resolution limit of the Athos gas detector.

In both measurements and simulations, we clearly see that the OK and helical configuration help to significantly reduce the saturation length. Furthermore, helical polarization achieves higher pulse energies for the two considered wavelengths, with increases of around 20% to 50%. For an effective and quantitative comparison between the different cases, we define, somewhat arbitrarily, the saturation pulse energy for each case as 60% of its maximum pulse energy. This definition applies without undulator taper, to avoid introducing additional complexities and keeping the analysis as robust as possible. We then define the saturation length for each case as the position along the undulator corresponding to the saturation pulse energy. We have tried other definitions, such as, for instance, defining the saturation point where the slope of the exponential gain drops below a certain factor. The chosen option gave similar but more consistent results (also in simulations) than the other attempted definitions.

**Figure 3:**
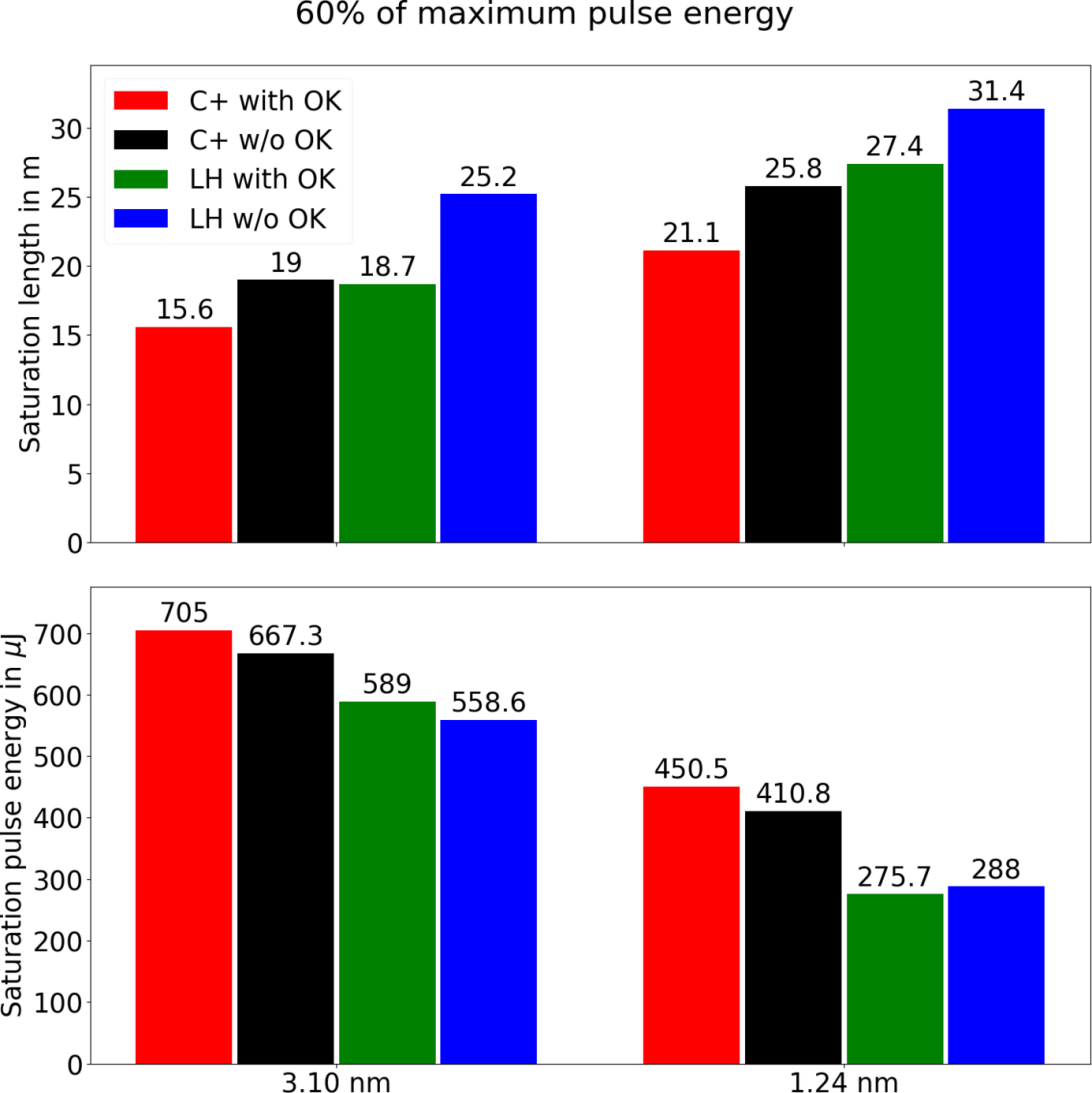
Comparison of saturation lengths (top) and saturation pulse energies (bottom) for the two measured radiation wavelengths and the four undulator configurations.

Fig. 3 ▸ compares the results for saturation length and saturation pulse energy between the various cases for the two radiation wavelengths. It is evident that the helical polarization reaches saturation faster and at higher pulse energies compared with the planar polarization, and that the OK helps (for both polarizations) to have a more compact undulator.

When combining helical polarization with OK we observe reductions of the saturation length by 38% and 33% for 3.10 nm and 1.24 nm, respectively, compared with the standard planar configuration without OK. Helical configuration and OK each account for about half of the total reduction, and the two enhancements can be combined effectively. The longer radiation wavelength (3.10 nm) shows a slightly stronger reduction of the saturation length than the shorter wavelength (1.24 nm). This is expected, since the OK is more efficient at longer FEL wavelengths, and since the benefit of the helical undulator configuration is higher for larger *K* values. The same effect is also borne out by the simulations described above. Our results concerning the OK are also in agreement with our earlier measurements (Prat *et al.*, 2021 ▸).

To compare the pulse energy results between helical and planar configuration, we average the saturation pulse energy between OK and no OK for each configuration. The saturation pulse energy increases by 20% for a radiation wavelength of 3.10 nm and by 53% for a radiation wavelength of 1.24 nm, when going from the standard planar to the helical undulator configuration. While this is qualitatively consistent with our simulations, the more pronounced improvement for the shorter wavelength is unexpected, since the benefit of the helical configuration should *a priori* be larger at higher *K* parameters. The discrepancy could be explained by the fact that the FEL gain curves were not measured with enough undulator modules for the shorter wavelength and planar configuration (or the longer wavelength and helical configuration), thus underestimating the saturation pulse energy for these cases.

## 5. Discussion and conclusion

To summarize, we have demonstrated an enhanced FEL performance when using helical undulators and OK with respect to the standard planar undulator configuration without OK: for radiation wavelengths of 1.24 nm and 3.10 nm, the saturation length is reduced by about 35% when using both effects and the pulse energy is increased by 20% to 50% for helical undulators. It is worth noting that the two effects, helical undulators and OK, can be combined effectively to reduce the saturation length considerably.

**Figure 4:**
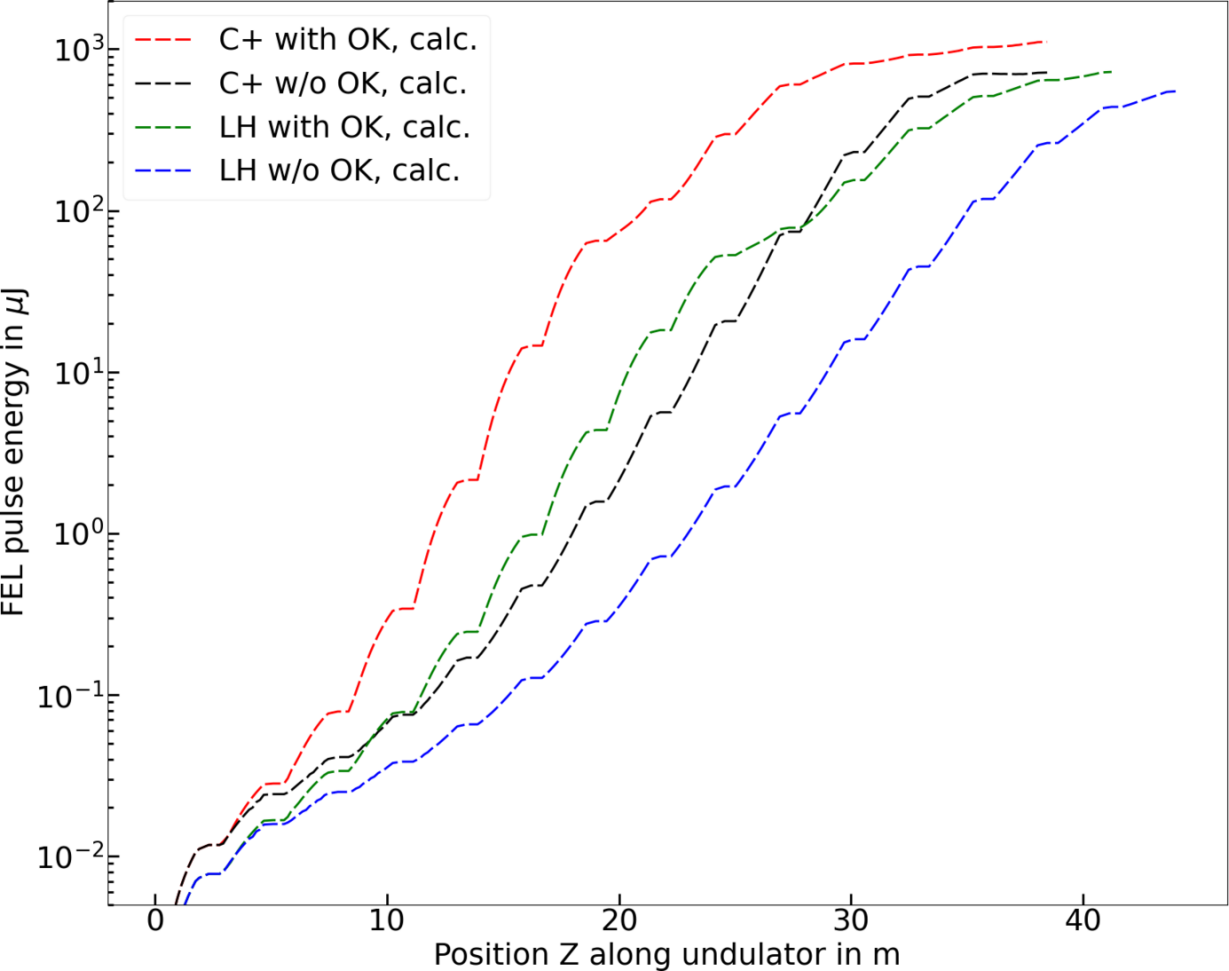
Simulated FEL gain curves for all combinations of helical (C+)/planar (LH) polarization and with/without OK for a radiation wavelength of 0.31 nm.

The results shown here for soft X-rays can be extrapolated towards shorter wavelengths. We have performed numerical simulations for a possible future beamline at SwissFEL, called Porthos (Schietinger, 2023 ▸), that would cover the wavelength range between 0.12 nm and 1.2 nm using a variable polarization undulator and intra-undulator chicanes. We have considered undulators with a period of 30 mm, an electron beam energy of 6 GeV, and a radiation wavelength of 0.31 nm (4.0 keV, corresponding to a *K* value of 1.92). The rest of the parameters are the same as used in the simulations shown earlier. For the OK cases, the chicanes before reaching FEL saturation are set to the optimum *R*_56_ given by equation (2), which for our parameters is 0.25 µm (equivalent to a delay of 0.41 fs), whereas the downstream chicanes are set to zero delay. The simulation results are shown in Fig. 4 ▸. We can observe a similar behavior as in the simulations and measurements for soft X-ray wavelengths reported above: the saturation length is reduced by around 30% when using the OK and helical undulators, and the pulse energy increases by around 35% with helical undulators.

The OK performance is better (worse) for lower (higher) values of δ/ρ, where ρ is the Pierce parameter (Bonifacio *et al.*, 1984 ▸). Therefore, reducing the energy spread is crucial to improve the efficiency of the OK effect. The ratio δ/ρ increases for compact facilities that employ relatively low electron beam energies and shorter undulator periods, as done for instance in the existing hard X-ray beamline of SwissFEL, called Aramis (Prat *et al.*, 2020 ▸). Therefore, improving the absolute energy spread is especially important for this type of compact facilities.

To conclude, our results pave the way for more compact, cost-effective and higher-power X-ray FEL facilities, thus making them more available and powerful for future scientific research. Future efforts should be directed towards improving the electron beam energy spread to increase the efficiency of the OK effect in reducing the FEL saturation length.

## Figures and Tables

**Table 1:** Electron bunch parameters and machine settings, during our measurements and used for calculations

Photon energy	400 eV	1000 eV
Wavelength	3.10 nm	1.24 nm
*K* parameter	3.50	1.92
Count of undulator modules used	8 to 11	10 to 14
Optimal initial delay	2.6 fs	1.2 fs
Energy spread	1.2 MeV	
Electron energy (γ)	6605.5	
Estimated slice emittance	300 nm	
Bunch length (used in simulation)	25 µm (20 µm)	
Bunch charge (used in simulation)	200 pC (160 pC)	
Peak current	2.4 kA	
Average β function	6 m	
